# Machine Learning-Based Prediction Models for Punching Shear Strength of Fiber-Reinforced Polymer Reinforced Concrete Slabs Using a Gradient-Boosted Regression Tree

**DOI:** 10.3390/ma17163964

**Published:** 2024-08-09

**Authors:** Emad A. Abood, Marwa Hameed Abdallah, Mahmood Alsaadi, Hamza Imran, Luís Filipe Almeida Bernardo, Dario De Domenico, Sadiq N. Henedy

**Affiliations:** 1 Department of Material Engineering, College of Engineering, Al-Shatrah University, Al-Shatrah 64007, Iraq; emadabed@shu.edu.iq; 2 Department of Civil Engineering, Najaf Technical Institute, Al-Furat Al-Awsat Technical University, Najaf Munazira Str., Najaf 54003, Iraq; marwah934@atu.edu.iq; 3 Department of Computer Science, Al-Maarif University College, Al-Anbar 31001, Iraq; alsaadi.m@uoa.edu.iq; 4 Department of Construction and Project, Al-Karkh University of Science, Baghdad 10081, Iraq; hamza.ali1990@kus.edu.iq; 5 GeoBioTec, Department of Civil Engineering and Architecture, University of Beira Interior, 6201-001 Covilhã, Portugal; 6 Department of Engineering, University of Messina, Villaggio S. Agata, 98166 Messina, Italy; dario.dedomenico@unime.it; 7 Department of Civil Engineering, Mazaya University College, Nasiriyah 64001, Iraq; ci.sadiq@mpu.edu.iq

**Keywords:** machine learning, fiber-reinforced polymer, flat slabs, punching shear capacity, gradient boosting regression tree

## Abstract

Fiber-reinforced polymers (FRPs) are increasingly being used as a composite material in concrete slabs due to their high strength-to-weight ratio and resistance to corrosion. However, FRP-reinforced concrete slabs, similar to traditional systems, are susceptible to punching shear failure, a critical design concern. Existing empirical models and design provisions for predicting the punching shear strength of FRP-reinforced concrete slabs often exhibit significant bias and dispersion. These errors highlight the need for more reliable predictive models. This study aims to develop gradient-boosted regression tree (GBRT) models to accurately predict the shear strength of FRP-reinforced concrete panels and to address the limitations of existing empirical models. A comprehensive database of 238 sets of experimental results for FRP-reinforced concrete slabs has been compiled from the literature. Different machine learning algorithms were considered, and the performance of GBRT models was evaluated against these algorithms. The dataset was divided into training and testing sets to verify the accuracy of the model. The results indicated that the GBRT model achieved the highest prediction accuracy, with root mean square error (RMSE) of 64.85, mean absolute error (MAE) of 42.89, and coefficient of determination (R^2^) of 0.955. Comparative analysis with existing experimental models showed that the GBRT model outperformed these traditional approaches. The SHapley Additive exPlanation (SHAP) method was used to interpret the GBRT model, providing insight into the contribution of each input variable to the prediction of punching shear strength. The analysis emphasized the importance of variables such as slab thickness, FRP reinforcement ratio, and critical section perimeter. This study demonstrates the effectiveness of the GBRT model in predicting the punching shear strength of FRP-reinforced concrete slabs with high accuracy. SHAP analysis elucidates key factors that influence model predictions and provides valuable insights for future research and design improvements.

## 1. Introduction

Steel corrosion greatly affects reinforced concrete structures, especially in harsh environments. In the United States, approximately one in seven bridges suffers from severe steel corrosion, resulting in annual expenditures of approximately USD 4 billion for maintenance and repairs [1,2]. These repairs not only incur high costs but also affect the performance of structures and cause public inconvenience due to the necessary closure of roads and buildings. These disturbances can cost up to ten times as much as corrosion repairs [2]. As a result, there is a growing interest in exploring alternatives to traditional steel reinforcement, with fiber-reinforced polymer (FRP) composites emerging as a promising solution. FRP, known for its cost-effectiveness, high tensile strength, lightness, and resistance to corrosion, stands out against conventional steel [3]. However, its adoption is limited due to its brittleness, lower elasticity, and higher initial costs [3]. As a result, extensive research is being conducted on the structural performance of RC elements using various FRP types, such as glass, carbon, aramid, and basalt FRP. These studies aim to ensure the safety and efficiency of RC structures with FRP reinforcement and to develop comprehensive design guidelines for its wider application [4,5,6,7,8,9,10].

Numerous experimental investigations have been conducted to decipher the factors influencing the structural performance of concrete beams, columns, slabs, and bridge decks reinforced with FRP. Key factors critically affecting the estimation of punching shear strength in FRP-reinforced concrete slabs include stiffness of FRP in bending and FRP–concrete bonding behavior [11], strength characteristics of concrete [12], dimensions of column and slab thickness [13], proportion of FRP reinforcement [14], arrangement of FRP within the slab [11], and span length to depth ratio [15]. The complexity of these parameters significantly complicates the use of traditional methods and formulas in accurately determining the punching shear capacity when FRP bars are incorporated.

The development of a punching shear strength formula for FRP bars is an ongoing task, evolving alongside the application of design codes. Studies are underway to enhance prediction formulas for FRP-reinforced concrete slabs, adapting methods from steel-reinforced slabs and considering the test-to-prediction ratio [11,15,16,17,18]. Furthermore, several studies have focused on using the modulus ratio (the ratio of the elastic modulus of FRP bars to steel bars) to enhance precision [19,20,21]. This approach has led to modified code formulas that incorporate the modulus ratio, drawing on standards like ACI 318-14 [22] and ACI 440-98 [20]. Drawing from a theoretical examination of the interaction between moment and shear in slabs, Theodorakopoulos and Swamy developed a model to determine the resistance to punching shear in slabs reinforced with FRP [23]. Meanwhile, Nguyen-Minh and Rovnak [24] introduced a prediction model grounded in fracture mechanics, effectively incorporating the impact of the span-depth ratio and dowel action. More recently, Ju et al. [25] formulated a predictive equation utilizing both deterministic and probabilistic approaches for estimating the punching shear strength in slab–column connections reinforced with both FRP and steel. Due to the diversity in the forms of these existing design equations, which are based on different parameters, there is a notable range in the accuracy of their predictions, with some showing low precision. Consequently, there is ongoing research interest in refining and producing more accurate and consistent design methodologies for assessing the punching shear capacity in FRP–RC slab–column connections.

In recent years, the application of machine learning (ML) in predicting the structural behavior of fiber-reinforced polymer (FRP) concrete slabs has gained great interest in the field of civil engineering [26,27,28,29,30]. A variety of studies have demonstrated the effectiveness of these ML models in enhancing the accuracy of predictions compared to traditional methods. One notable development is the development of a hybrid ML model that integrates the capabilities of a least squares support vector machine (LS-SVM) and the firefly algorithm [30]. This model has shown significant improvement in predicting the shear capacity of FRP-reinforced panels, significantly reducing prediction errors and outperforming traditional methods. To further contribute to this field, the joint reinforcement tree model, as developed in one study, excelled in predicting the behavior of slab–column connections with FRPs [31]. This model has high prior accuracy and low error rates and consistently exceeds the accuracy of ML and other traditional models. Another innovative approach involves the use of adaptive reinforcement in ML to enhance the prediction of shear strength in FRP-reinforced concrete slabs [28]. This technique has proven to be more accurate than existing models and codes. Additionally, it includes SHapley Additive exPlanations (SHAP) analysis to better understand the factors that influence its forecasts and to provide some mechanical interpretation of the resulting outcomes. The use of XGBoost in ML algorithms was also an important step forward [29]. It has demonstrated superior performance in accurately predicting the punching shear strength of FRP-RC slabs, showing higher accuracy and reliability than other ML models and existing design codes. Artificial neural networks (ANNs) have also been used in this field [32]. A study analyzing 164 tests found that the ANN model with 6 input neurons, 15 neurons in the first hidden layer, 5 neurons in the second hidden layer, and 1 output neuron is highly accurate, with an impressive R value and mean absolute percentage error (MAPE), thus outperforming existing models. This research also led to the development of a practical graphical user interface for industry applications. Finally, the hybrid “gray box” model, which integrates modulated pressure field theory (MCFT) with a machine learning-assisted approach (genetic programming), represents a major advance [27]. This model, which was tested using a database of 154 experiments, showed performance enhanced by an efficient correction equation, exceeding the accuracy of five other experimental models.

In this study, we have developed a gradient boosting regression tree (GBRT) model [33] tailored for predicting the shear strength of FRP slabs. This model strategically builds additional trees, aiming to reduce prediction errors from the foundational models. The GBRT’s boosting technique is especially effective in complex situations, creating an ideal series of trees. This is particularly advantageous in cases with few samples but many input features. The model excels in comprehending the complex dynamics and non-linear behaviors of FRP slabs, thereby boosting prediction accuracy. Moreover, the GBRT method not only precisely predicts the shear strength from various features but also identifies and prioritizes the significance of each feature for a more detailed examination. 

The aim of this study is to use the GBRT model with a much larger dataset than previous models to enhance accuracy in predicting the behavior of fiber-reinforced polymer (FRP) reinforced concrete panels. This expanded dataset approach was designed to overcome limitations encountered in previous studies, which often relied on smaller, less diverse data. By using a more comprehensive dataset, we expect to not only improve the accuracy and reliability of predictive models but also improve ML algorithms through more robust training and validation. Furthermore, this approach facilitates comprehensive comparison with previous studies, showcasing advances in predictive capabilities while enhancing the generalizability of the results. A larger dataset will also likely reveal new insights and trends, deepening our understanding of the factors that influence FRP-reinforced concrete slabs. In addition to providing more accurate, reliable, and universally applicable predictive models, this study also aims to develop a user-friendly graphical user interface (GUI) tool. This tool will be accessible via the web, allowing engineers to easily apply our findings and predictive models in their work, significantly advancing the current understanding and application in this field.

## 2. Materials and Methods

**Figure 1:**
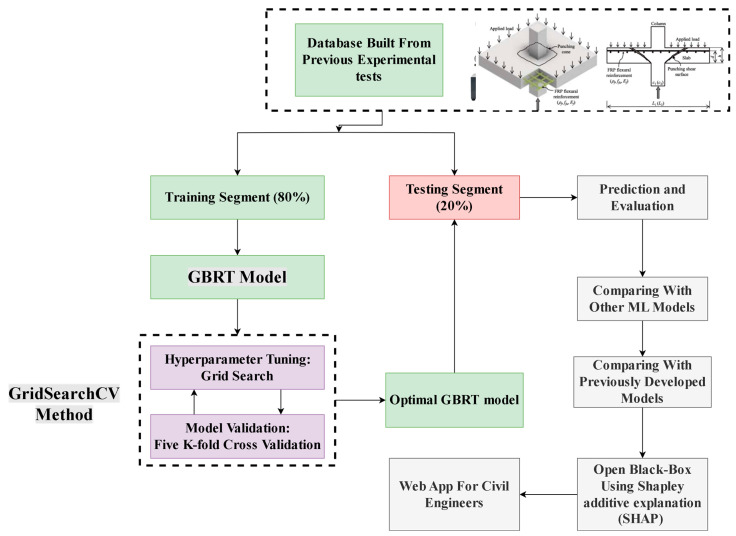
Research methodology.

Figure 1 showcases the entire methodology devised for our approach. The first action taken was the creation of a database consisting of reinforced FRP concrete slabs. This database was then processed using specific engineering parameters and filtering methods. Following this, the dataset was divided into two distinct sections: one for training purposes and the other for testing. Based on the guidelines from previous research [34], it is recommended that each feature contain between 10 and 30 samples. Our database contains 238 records for each feature, which exceeds the recommended number from the previous study. Additionally, the same research recommended a data partition ratio of either 65:35 or 80:20. In our study, we used the 80:20 splitting technique. Utilizing the training data, the GBRT model underwent training that included the application of a five-fold cross-validation technique, integral in determining the most effective hyperparameters for the model during this phase. The next phase involved testing the model’s effectiveness on the testing set after optimizing for hyperparameters and comparing its performance with other ML models, including a black box model like KNN and a white box model such as Lasso regression. If the model’s performance was deemed satisfactory, it could be considered a final predictive model. In the concluding phase, the predictive accuracy of the GBRT model was compared with previously developed empirical equations using well-established performance measurement methods. Additionally, the SHAP algorithm is utilized in this research as an effective tool to open the black box of GBRT models and to determine the effect of each variable on model prediction. Finally, a graphical user interface (GUI) was created to enable civil engineers to utilize the model, enhancing their ability to design and verify FRP-reinforced concrete slabs.

### 2.1. Previously Developed Equations

The code equations for steel-reinforced slabs in ACI 318-05 [35] and BS 8110-97 [36], provided below, have been modified by researchers to account for the lower elastic modulus of FRP reinforcement. This modification aims to assess the punching shear capacity of FRP-reinforced slabs. The following values are given in metric units: dimensions in mm, strength in N, stresses in MPa, and elastic moduli in GPa.

In the absence of flexural reinforcement as a significant factor, the punching shear resistance of an internal square column steel-reinforced flat slab is given by ACI 318-05 [35] as follows:

(1) $$V_{C}=0.33\sqrt{f_{c}^{\prime }}b_{o}d$$

where *d* is the average effective slab flexural depth; *b*_o_ is the perimeter at the critical section, which is situated 0.5 *d* from the column face; and $f_{c}^{\prime }$ is the required cylinder compressive strength of concrete.

For steel-reinforced slabs, *V_c_* is computed as follows in BS 8110-97 [36]:

(2) $$V_{C}=0.79{\left(100\rho_{s}\right)}^{1/3}{\left(f_{cu}/25\right)}^{1/3}(400/d{)}^{1/4}b_{p}d$$

where *ρ*_s_ is the steel reinforcement ratio; *f*_cu_ is the cube concrete compressive strength; and *b*_p_ is the rectangular critical perimeter that is 1.5 *d* from the column face, independent of the form of the column. The following FRP slab adjustments have been suggested based on these equations.

El-Ghandour, Pilakoutas, and Waldron [19] added the term (*E*_f_/*E*_s_)^1/3^ to the ACI code equation, where *E*_f_ and *E*_s_ stand for modulus of elasticity for steel and FRP, respectively. Therefore, for FRP-reinforced slabs, Equation (3) becomes:

(3) $$V_{C}=0.33\sqrt{f_{c}^{\prime }}{\left(E_{f}/E_{s}\right)}^{1/3}b_{o}d$$

As a modification of the BS 8110 equation, Matthys and Taerwe [15] developed the following equation for two-way slabs reinforced by FRP bars or grids:

(4) $$V_{C}=1.36{\left[100\rho_{f}\left(E_{f}/E_{s}\right)\right]}^{1/3}f_{cm}^{1/3}(1/d{)}^{1/4}b_{p}d$$

where *f*_cm_ stands for the average compressive strength of concrete after 28 days in cylinders. 

Additionally, an empirical equation based on Equation (4) was suggested by Ospina, Alexander, and Cheng [11]. It is given by:

(5) $$V_{C}=2.77{\left(\rho_{f}f_{c}^{\prime }\right)}^{1/3}{\left(E_{f}/E_{s}\right)}^{1/2}b_{p}d$$

The scaling effect on the punching of slabs with FRP reinforcement has been excluded from Equation (5) because it was reported that this effect was not evident based on the available FRP test results [11]. To produce better results, the effect of the modular ratio *E*_f_/*E*_s_ is considered as the square root instead of the cube root.

Also, to estimate the punching shear capacity of both steel and FRP-reinforced concrete flat slabs, El-Gamal et al. [37] suggested altering the ACI 318-05 punching shear equation by multiplying it by a new factor *α*. The form of the modified equation is:

(6) $$V_{C}=0.333\sqrt{f_{c}^{\prime }}b_{oACI}d\alpha$$

(7) $$\alpha =0.5(\rho E{)}^{1/3}\left(1+\frac{8d}{b_{oACI}}\right)$$

Finally, Ju et al. [25] developed a probability distribution with a significant unknown factor using Monte Carlo Simulation (MCS) to ensure the lowest likelihood of failure. The design strength equation below was determined using a probabilistic technique with 95% confidence:

(8) $$\begin{array}{r} V_{c}=2.3{\left[\rho_{f}\left(E_{f}/E_{s}\right)f_{c}^{\prime }\right]}^{1/3}{\left(d/b_{0,0.5d}\right)}^{1/2}b_{0,0.5d}d \end{array}$$

### 2.2. Gradient Boosting Regression Tree (GBRT)

The CART system (Classification and Regression Trees) was introduced by Breiman et al. [38]. Regression and classification models can both be implemented with CARTs [39,40,41]. The decision trees used in these two models are binary trees, which are made using recursive techniques. The main focus of this study is the GBRT approach, which combines the Gradient Boosting and CART methods developed by He et al. [42]. More accurate in prediction than other AI systems, the CART system is acclaimed for its ability to describe nonlinear interactions without requiring prior knowledge of variable probability distributions [42]. In this study, the Gradient Boosting approach transforms ineffective learners into effective ones, while the CART system is used to build regression trees for weak learners. The system is trained with weak learners to improve the previously learned predictions, reducing forecasting errors and boosting reliability.

The GBRT approach sets the initial value of $F_{0}(x)$ as follows:

(9) $$F_{0}(x)=\underset{c}{argmin}\sum_{i=1}^{N}\;L\left(y_{i},c\right)$$

A loss function is shown by L(. ). When calculating the residual prediction at iteration *m*, where *m* = 1,…, M, the following equation may be utilized to obtain the negative gradient value of the loss function in the present framework:

(10) $$r_{m,i}=-{\left[\frac{\partial L\left(y_{i},F\left(x_{i}\right)\right)}{\partial F(x)}\right]}_{F(x)=F_{m-1}(x)},i=1,.,N$$

Since each tree is predicted to have *J_m_* splits, this regression tree divides the input area into *J_m_* disjoint regions *R_m,1_*,…, *R_m,Jm_*. Each tree then projects a value *c_m,j_* for area *R_m,j_*. The following formula may be minimized to obtain *c_m,j_*:

(11) $$c_{m,j}=\underset{c}{argmin}\sum_{x_{i}\in R_{m,j}}\;L\left(y_{i},F_{m-1}(x)+c\right)$$

The mth regression tree of the updated algorithm’s linked leaf node area, *F_m_*(*x*), or *R_m,j_*, where *j* = 1, 2,…, *J_m_*, is determined as follows:

(12) $$F_{m}(x)=F_{m-1}(x)+\sum_{j=1}^{J_{m}}\;c_{m,j}I\left(x\in R_{m,j}\right)$$

**Figure 2:**
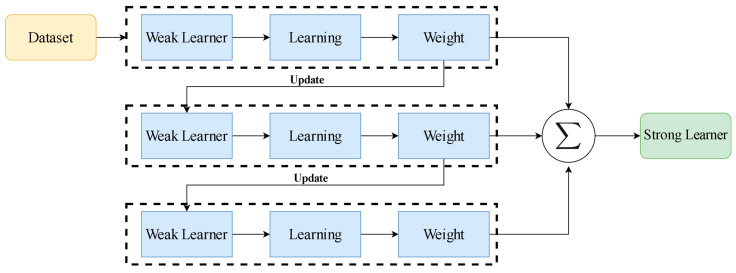
Workflow of gradient boosting for regression trees (GBRT).

If *x* ∈ *R_m,j_*, then *I* equals one; otherwise, it equals zero [43]. *J_m_* also indicates the number of leaf nodes in the mth regression tree. After that, the design is updated. Figure 2 presents an overview of the GBRT methodology.

### 2.3. K-Nearest Neighbors

A non-parametric ML approach called K-Nearest Neighbors makes no assumptions about the decision boundaries separating the failure modes. Using this strategy, a sample is considered to belong to a failure mode if it is one of the *K* most comparable samples in the feature space. Next, we estimated the conditional probability for *x* in failure mode *f* as follows:

(13) $$P_{r}(x)=P_{r}(Y=f|X=x)=\frac{1}{K}\sum_{j\in N_{K}}\;I\left(y_{i}=f\right)$$

In the training data, *K* points that are closest to observation, *f*, are represented by *N*_*K*_.

### 2.4. Least Absolute Shrinkage and Selection Operator (LASSO)

Tibshirani proposed the LASSO model algorithm in 1996 [44]. The basic idea is to minimize the sum of squared residuals while adhering to a constraint that sets a maximum value for the sum of the absolute values of the regression coefficients. This method allows us to produce certain regression coefficients that are precisely equal to zero, which makes it possible to build a more reliable model with more potent explanatory power. Compared to linear regression, this method is similar to Ridge regression in that regularization coefficients are included. But LASSO regression uses L_1_ regularization, which sets it apart from Ridge regression [45]. The expression for the loss function is:

(14) $$J=\frac{1}{n}\sum_{i=1}^{n}\;{\left(f\left(x_{i}\right)-y_{i}\right)}^{2}+\lambda {∥w∥}_{1}$$

where *λ*‖*w*‖_1_ is the L_1_ regularization factor.

### 2.5. Cross-Validation

Many researchers used the standard method of data splitting, which includes dividing the dataset into three subsets—training, validation, and testing—to evaluate the performance of their ML model. The validation set monitors the model’s performance, while the training set is utilized to complete the learning process. Lastly, the model is put to the test by running it through a testing set of samples it has never seen before in order to assess its extrapolation abilities [46]. The previously mentioned process can lead to an inadequately trained model due to dataset size reduction resulting from the division into three subsets. Therefore, especially for small datasets, cross-validation is a commonly used technique to prevent excessive reduction of the training set [46]. There are various approaches to implementing cross-validation, but the most popular one involves validating the model by excluding random inputs. In this study, K-fold cross-validation was employed. This resampling method, known as K-fold cross-validation, divides the data into K subsets: K−1 for training and one subset for validation. 

### 2.6. Hyperparameter Tuning

**Figure 3:**
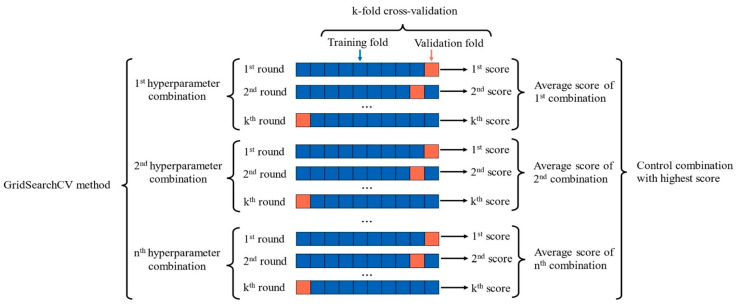
Hyperparameter optimization using GridSearchCv.

Building a reliable ML model requires a fundamental step, which is tuning the hyperparameters of the model. Hyperparameter tuning is useful to increase the model’s adaptability to new data and reduce the problem of overfitting [47]. In addition, the model’s accuracy is increased through the process of finding the best hyperparameters [48]. Grid search and random search optimization methods are the most popular hyperparameter tuning procedures that have been used to automate hyperparameter selection and prevent manual tuning [49]. What distinguishes these methods from each other is the range of possible values that are evaluated during the research process. While grid search evaluates all possible values of hyperparameters within a predefined range, random search techniques randomly select different values of hyperparameters for a given number of iterations [49]. The Scikit-Learn package in Python (3.7) [50] was used to explore possible values of hyperparameters using the grid search technique with five-fold cross-validation (GridSearchCV). Figure 3 depicts the five-fold cross-validation used in this work for training and hyperparameter selection of the model.

### 2.7. Performance Measure

To evaluate the performance of the developed models analytically, the following statistical error parameters were utilized: Mean Absolute Error (MAE), Root Mean Square Error (RMSE), correlation coefficient (*R*), and coefficient of determination (*R*^2^).

The MAE is calculated as follows:

(15) $$\;\mathrm{MAE}\;=\frac{\sum_{i=1}^{N}\;\;\left|P_{i}-O_{i}\right|}{N}$$

The RMSE is defined as follows:

(16) $$\mathrm{RMSE}\;=\sqrt{\frac{1}{N}\sum_{i=1}^{N}\;\;{\left(P_{i}-O_{i}\right)}^{2},}$$

The correlation coefficient (*R*) is determined by the following equation:

(17) $$R=\frac{\sum_{i=1}^{N}\;\;\left(P_{i}-P_{m}\right)\left(O_{i}-O_{m}\right)}{\sqrt{{\left(P_{i}-P_{m}\right)}^{2}}\sqrt{{\left(O_{i}-O_{m}\right)}^{2}}}$$

The coefficient of determination (*R*^2^) is computed as follows:

(18) $$R^{2}=1-\frac{\sum_{i=1}^{N}\;\;{\left(O_{i}-P_{i}\right)}^{2}}{\sum_{i=1}^{N}\;\;{\left(O_{i}-O_{m}\right)}^{2}}$$

where *N* is the number of data points, $O_{m}$ is the mean of observed values, and $P_{m}$ is the mean of predicted values. In this case, $O_{i}$ stands for the measured values and *P*_*i*_ for the expected values. The relative correlation between the observed and expected values is expressed as the correlation coefficient (*R*). The range of *R* values spans from −1 to 1. A direct linear connection between the measured and anticipated values is shown by a *R* value that is close to 1. *R*, however, may not always accurately reflect the performance of the model, especially when the data range is large and the data points are dispersed around the mean. As a result, the coefficient of determination, or *R*^2^, is a more objective estimate and useful tool for assessing how well the model performs. The discrepancy between the measured and predicted values is evaluated by the MAE and RMSE, with values close to zero indicating more accuracy.

## 3. Database Used

**Figure 4:**
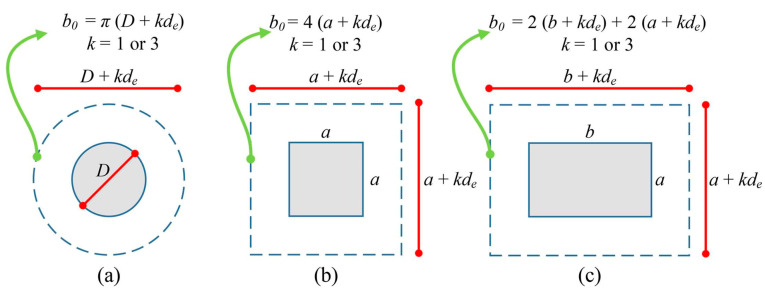
Process of calculating the perimeter of the critical section in RC slabs, *b_0_* for: (**a**) circular; (**b**) square; and (**c**) rectangular cross-sections [32].

For this investigation, a dataset of 238 experimental observations on FRP-reinforced concrete slabs under punching shear was gathered from a range of literature sources [11,14,15,16,18,20,24,37,51,52,53,54,55,56,57,58,59,60,61,62,63,64,65,66,67,68,69,70,71,72,73,74,75,76,77,78,79,80,81,82,83,84,85,86,87,88,89]. It is worth mentioning that most of the experimental data included in our database was also part of the comparative study conducted by [90]. The findings from previous work [32] revealed that there is a significant impact of a number of critical variables on the punching shear of FRP-reinforced concrete slabs. These variables include the cross-section area of the column (*A*), the perimeter of the critical section in the RC slab (*b*_0_), which can be calculated based on 0.5*d_e_* and 1.5*d_e_* and is named *b*_0,0.5*de*_ (*k* = 1) and *b*_0,1.5*de*_ (*k* = 3), as demonstrated in Figure 4. The effective depth of the RC slab is denoted as de, while *f_c_* represents the compressive strength of the utilized concrete in the RC slab. The modulus of elasticity and the reinforcement ratio of the steel and FRP rebars are respectively indicated as *E_r_* and *ρ_r_*. As a result, ML models that estimate the shear strength of FRP-reinforced concrete slabs are built using these discovered parameters as their foundation.

The dataset encompasses a range of values for each parameter: the cross-section area of the column (*A*) ranging from 6.25 to 1587.5 cm^2^, the perimeter of the critical section in the RC slab (*b*_0,0.5*de*_) ranging from 280 to 2470 mm, and (*b*_0,1.5*de*_) ranging from 640 to 4608 mm. The effective depth of the RC slab (de) ranges from 36 to 284 mm, the compressive strength of the utilized concrete in the RC slab (*f_c_*) ranges from 22.16 to 179 MPa, the reinforcement ratio (*ρ_r_*) ranges from 0.13 to 3.76%, the modulus of elasticity of the steel and FRP rebars (*E_r_*) ranges from 28.4 to 230 GPa, and the ultimate shear strength (Vu) ranges from 24 to 1600 kN. Square and circular columns are among the test specimens. They consist of flat slab-column connections constructed of FRP-reinforced concrete without drop panels, column capitals, or shear reinforcement.

**Figure 5:**
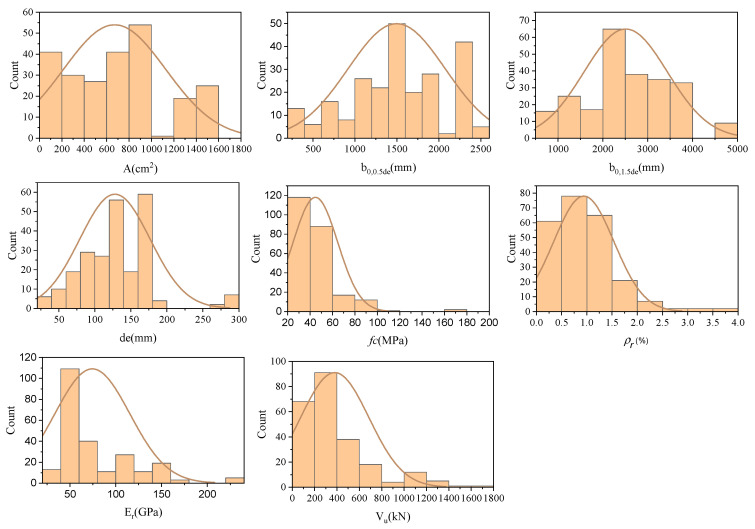
Histograms of input and output variables.

**Figure 6:**
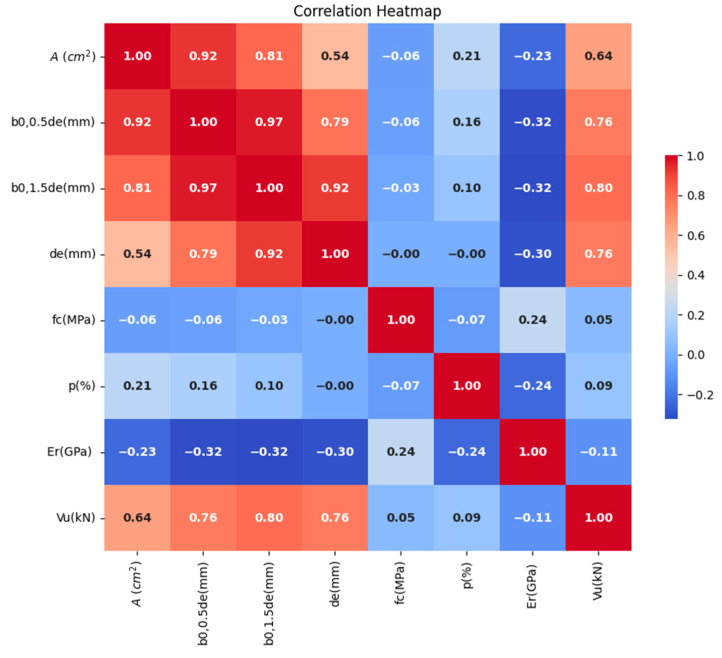
Pearson correlation between input and output variables.

The statistical parameters summarizing the properties of the variables in the database are presented in Table 1, while Figure 5 illustrates the distribution of each parameter. Additionally, the correlation matrix plot in Figure 6 displays the association between shear capacity and the six input parameters. This figure graphically represents the pairwise correlations between the parameters, each associated with a corresponding correlation coefficient. It is evident that while parameters *f_c_*, *ρ_r_*, and *E_r_* show a weaker connection with shear strength, parameters *b*_0,0.5*de*_, *b*_0,1.5*de*_, de, and *A* exhibit a significant correlation with shear strength.

## 4. Model Results

### 4.1. Cross-Validation Results

**Figure 7:**
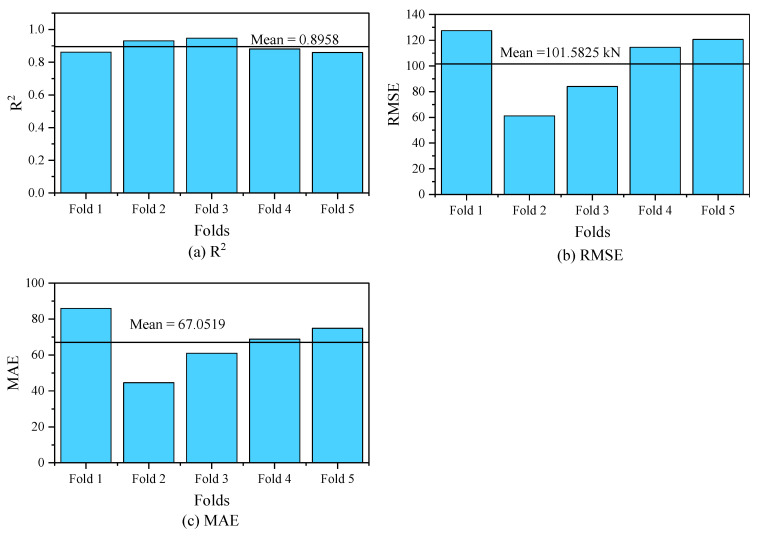
Five-fold cross-validation results for evaluation metrics: (**a**) R^2^, (**b**) RMSE, and (**c**) MAE.

The obtained results of K-fold cross-validation used to assess the performance of the GBRT model are illustrated in Table 2 and Figure 7. Across all the metrics used to test the performance, the model displays strong consistency and reliability. A low standard deviation and an average of 0.8958 indicate stability throughout the five folds. Also, we can notice that the R^2^ values for five folds are mostly above 0.85, demonstrating its resilience in capturing the variance in the data. The RMSE, which averages 101.5825 kN with a standard deviation of 27.2149 kN, highlights the model’s accuracy and stable error rate. Bar charts visually support these findings by showing values tightly clustered around the mean, indicating consistent predictive power. Similarly, the MAE shows an average error magnitude of 67.0519 kN. Overall, the cross-validation performance demonstrates the model’s ability to make accurate predictions and its potential for practical application. 

### 4.2. Performance Comparison with Other ML Models

**Figure 8:**
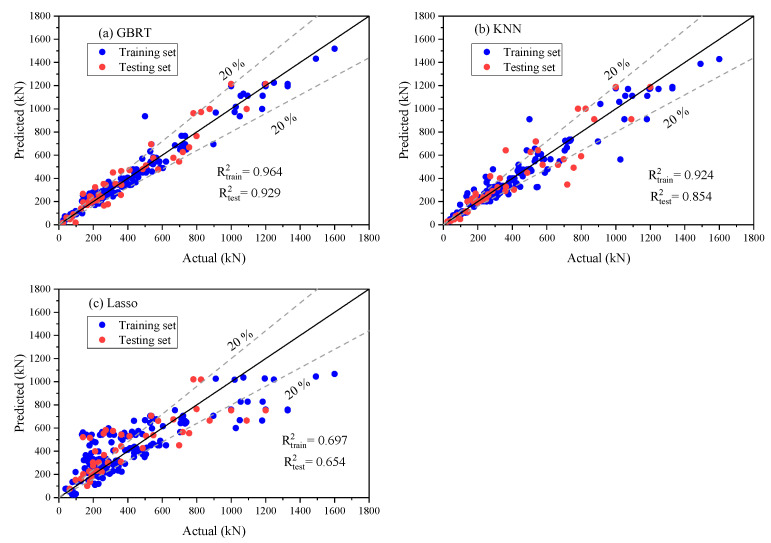
Scatter plot for predicted vs. actual values for training and testing sets using (**a**) a GBRT, (**b**) KNN, and (**c**) LASSO.

As displayed in Figure 8a–c, the results of our developed GBRT model showed minimal scattering around the ideal line between the actual and predicted shear strengths for both the training and testing datasets. Also, Table 3 presented an analytical investigation of statistical error parameters for training and testing datasets to support the validation of the generated model. In Table 3, the outcomes from the developed Lasso and KNN methods were also evaluated against our proposed model. Previous authors have suggested that an R value greater than 0.8 indicates a strong correlation between observed and predicted values. Note that the observed and predicted values may not match even if R is near one. Instead, they just vary in a similar way. The coefficient of determination (R^2^) can be employed to address this limitation. For accurate results, R^2^ values should be close to 1. The accuracy achieved by GBRT for both training and testing was better compared to Lasso and KNN, as we can notice in Table 3. For all these data, when comparing the performance of the GBRT model with the KNN and Lasso models, the GBRT model increased R^2^ values by 4.19% and 39.17%, respectively. Additionally, it lowered RMSE values by 26.53% compared to KNN and by 62.12% compared to Lasso. The GBRT model also achieved a reduction in MAE values by 19.09% compared to KNN and by 65.80% compared to Lasso. Notably, while the performance of the KNN and LASSO models was found reasonable, KNN demonstrated superior results over LASSO. The lower performance of the LASSO model can be linked to its inability to accurately predict phenomena that are highly nonlinear or involve multiple criteria.

**Figure 9:**
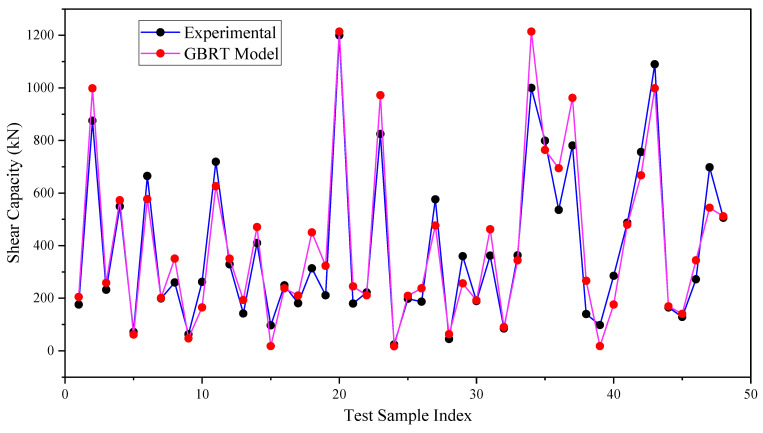
Comparison of experimental results and GBRT model predictions for the testing dataset.

Figure 9 further emphasizes the effectiveness of our suggested model through plotting. The actual shear resistance versus experimentally observed shear strength for the testing databases are shown in this figure. This figure demonstrates how closely the suggested models’ predictions match the strength seen through experimentation. Despite not being utilized for model training, the testing database predicted data points are close to the experimental points, demonstrating good prediction capacity. This illustrates our suggested models’ capacity for generalization. As shear strength values rise, the data exhibit greater dispersion, primarily because there are fewer observations available.

### 4.3. Performance Comparison with Previously Developed Models

**Figure 10:**
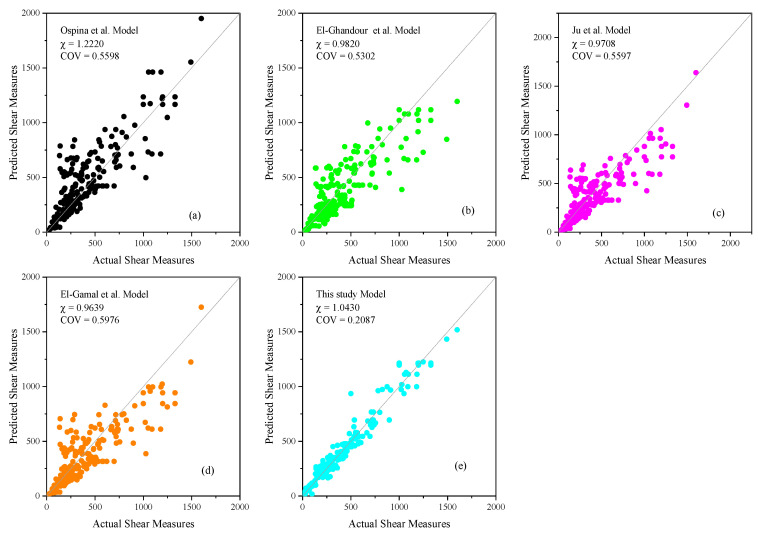
Comparison of different model scatter plots for (**a**) Ospina et al. [11], (**b**) El-Ghandour et al. [19], (**c**) Ju et al. [25], (**d**) El-Gamal et al. [37], and (**e**) this study model.

Figure 10a–e depict the experimental versus predicted shear capacities using models by Ospina et al. [11], El-Gamal et al. [37], El-Ghandour et al. [19], Ju et al. [25], and the proposed GBRT model. The equity solid line in these figures represents a perfect match between the experimental and predicted responses. Table 4 evaluates these models based on the mean, standard deviation (STD), and coefficient of variation (COV) of the pred/exp ratio (χ). The proposed GBRT model shows the best performance with a mean χ of 1.0430, a standard deviation of 0.2177, and a COV of 0.2087, indicating high accuracy and stability. The close clustering of data points around the mean in the corresponding figure further confirms the model’s reliable predictive power. In contrast, the Ospina et al. [11] model tends to overestimate shear capacity, as indicated by a high mean χ of 1.2220 and a high standard deviation of 0.6841, resulting in a COV of 0.5598. This model’s scatter plot shows a wider spread of data points, reflecting less consistent predictions. The El-Gamal et al. [37] model has a mean χ of 0.9639, a standard deviation of 0.5760, and a COV of 0.5976, indicating moderate accuracy but less stability. Similarly, the El-Ghandour [19] model, with a mean χ of 0.9820, a standard deviation of 0.5206, and a COV of 0.5302, shows relatively better performance among the existing models. The Ju et al. [25] model also performs moderately well, with a mean χ of 0.9708, a standard deviation of 0.5433, and a COV of 0.5597. The scatter plots for these models show varying degrees of data clustering around the mean, reflecting their predictive capabilities.

### 4.4. Reliability Analysis

For assessing structural reliability, relying solely on basic descriptive statistics of χ might not provide sufficient information about the reliability of a design proposal. An χ value of 2 indicates a more severe issue than a χ value of 0.5, a nuance that is not captured by traditional statistical analysis. To address this, the Demerit Point Scale (DPS) Methodology is used, which assigns penalty points to different ranges of χ values [91]. This methodology divides the χ ratio into sections, each associated with a specific penalty, as outlined in Table 5. The total demerit point score for each model is calculated by multiplying the percentage of χ values in each range by the corresponding penalty points, summing these products, and dividing by 100. This score ranges from 0 to 10, with lower scores indicating better performance. Table 6 details the χ ranges, their penalties, and the percentage of specimens in each range. The overall performance of each model is then evaluated using a cumulative penalty score. Compared to existing models, the GBRT model shows the highest percentage (65.13%) of predictions within the appropriate safety range (0.85 < χ ≤ 1.15) and the lowest percentage (0.42%) in the dangerous and extra dangerous ranges. Thus, the GBRT model has the lowest total demerit point score, indicating higher reliability. In contrast, the El-Ghandour et al. [19] model is the most conservative, with 5.88% of predictions in the extremely conservative range. The Ospina et al. [11] model is the least reliable, with 38.65% of predictions falling in the dangerous and extra dangerous ranges. This analysis highlights the GBRT model’s superior predictive accuracy and reliability compared to other models. 

### 4.5. Shap Analysis

To understand the importance of each input factor, we used SHAP (SHapley Additive exPlanations). This method, created by Lundberg and Lee in 2017 [92], calculates how much each input affects the result. It uses a game theory concept called Shapley values. SHAP looks at all possible combinations of inputs to find the average effect of each one. The higher the SHAP value, the more important the input is. In short, SHAP helps us to see which inputs matter most for the results.

**Figure 11:**
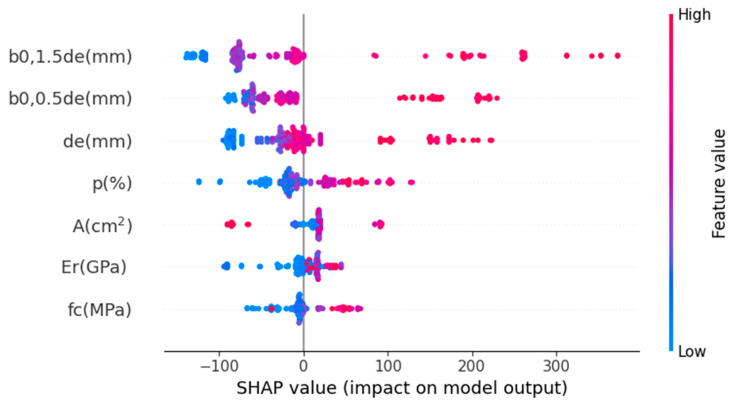
SHAP summary plot for the GBRT model.

Summaries of the related attribute patterns and SHAP value distributions for individual features are displayed in Figure 11. The input variables are shown on the *y*-axis of the summary plot, arranged by significance, and the SHAP values are shown on the *x*-axis. The dots represent the database instances, with their color indicating intensity (ranging from blue to red). The *x*-axis displays the range of predictions for each variable, as shown by the SHAP values, highlighting the range of input variable magnitudes from blue to red. Utilizing it can help one better understand how each unique attribute affects the prediction of the dependent variable. When a feature’s magnitude is high, it can significantly impact the dependent variable’s prediction, as indicated by its position on the right side of the plot and its high SHAP value. According to Figure 11, the critical section perimeters (*b*_0,1.5*de*_ and *b*_0,0.5*de*_) have the greatest magnitude of SHAP values and the greatest impact on the model predictions. The critical section perimeters can also be considered as a vital variable influencing slab punching shear capacity [93]. Notably, the slab effective depth *d_e_* emerges as the third most crucial feature, aligning with findings from reference [13]. It can be seen that the most important features that strongly affect the shear strength of a flat slab are the geometric parameters. This is because the behavior of the slab, especially its failure mode (bending or shearing), depends on the cross-sectional properties, the slab’s depth, and the load point’s position. In addition, the material properties of the concrete also play an essential role when the slab collapses due to shear. Despite the limited contribution of the dowelling action of FRP bars to punching shear capacity, heightened values of *ρ* and Er result in decreased deformation and crack width. This, in turn, enhances aggregate interlock and increases the shear resistance of concrete [13,16,94]. In summary, SHAP analysis is a valuable tool for enhancing our understanding of the proposed models, particularly regarding the influence of input features on the predicted parameter. The effect of input parameters can be analyzed in the context of traditional mechanical backgrounds to increase confidence in ML-based predictions, which are sometimes viewed as “black-box” processes.

### 4.6. Model Uncertainty Analysis

**Figure 12:**
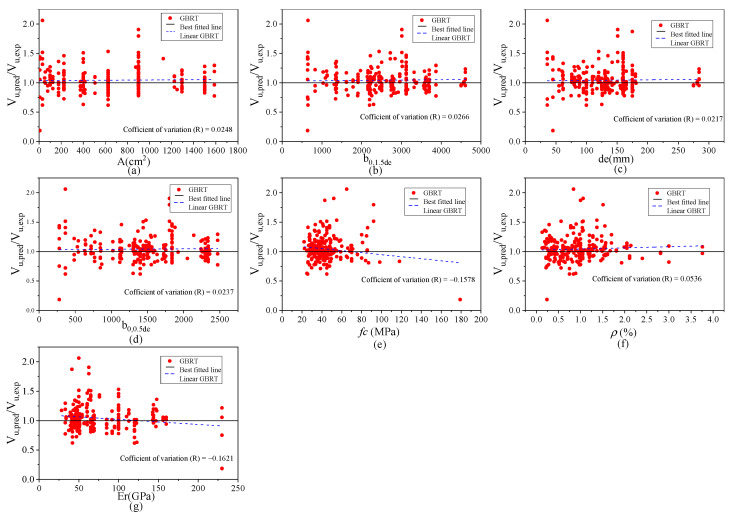
Trend analysis between the predictors and the ratio of predicted/actual based on the GBRT model: (**a**) A (cm^2^), (**b**) *b*_0,1.5*de*_, (**c**) *d_e_*, (**d**) *b*_0,1.5*de*_, (**e**) *f_c_* (MPa), (**f**) *ρ_r_* (%), (**g**) Er (GPa).

Correlation and regression analysis [95] were performed on datasets of model uncertainty observations to determine the sensitivity of critical shear capacity design parameters to various variables. According to Franzblau [96], correlations are categorized as very weak (0 to ±0.2), weak (0.2 to ±0.4), and moderate (0.4 to ±0.6). Scatter plots of model uncertainties versus factors affecting the shear capacity of flat slabs are presented in Figure 12. The calculated linear correlation coefficients are also summarized in Figure 12, showing the degree of correlation between model uncertainty (predicted/actual) and model input parameters. A reliable shear prediction model for FRP-reinforced concrete flat slabs should exhibit very weak or no correlation with design parameters that influence shear resistance. This indicates that the model adequately accounts for the influence of design parameters, resulting in insignificant systematic trends. As shown in Figure 12, there is no notable bias or trend toward these variables, and the prediction accuracy for the ultimate shear strength using the GBRT-based relationship appears robust. Modifications to the model’s application are required for situations with significant trends, particularly in areas of potentially dangerous or uneconomic performance. These modifications might involve restricting the model’s use to specific scenarios, implementing calibration procedures to include sufficient safety components in the event of dangerous performance, or adding a calibrated resistance adjustment factor for uneconomic performance. Many of these correction processes are now included in various design standards.

### 4.7. Graphical User Interface

The models developed in this study provide a practical ML solution for estimating the FRP flat shear strength under different input parameters. To ensure the predictions are computationally efficient, user-friendly, and accurate, a graphical user interface has been created. This interface is available to all users through the following accessible link: https://frpflatslabshear-bhev7apr89guhlsgoybpjy.streamlit.app/. 

Using movable digital sliders, the user interacts with the web application to specify the main input parameters (*A*, *b*_0,0.5*de*_, *b*_0,1.5*de*_, *d_e_*, *f_c,_ ρ_r_*, and Er) during the first stage of prediction through the designed GUI. These parameter ranges correspond to those found in the datasets on which the ML models were trained. The screen interface displays both the computed values and the parameters supplied by the user. Most importantly, the code runs automatically whenever the input variables are changed. The prediction process is noteworthy for being extremely quick, typically taking only a few seconds.

Without requiring the complex computations typically associated with mechanics-driven models, this graphical interface tool can be invaluable for engineers and practitioners seeking more accurate prediction outcomes. It significantly enhances efficiency and accuracy. The program’s intuitive user interface (UI) makes it a practical tool, saving time and effort when predicting the FRP flat shear strength under different input parameters. 

One of the main advantages of this application is its ability to be accessed through a web browser using the supplied URL. Because of its web-based accessibility, the software tool can be deployed on local computing devices without the need for program installation.

## 5. Recommendation for Future Work

Our current study focuses on predicting the punching shear capacity of FRP flat slabs, ranging from 24 to 1600 kN, based on large-scale experimental results from previous literature using the GBRT model. Since the GBRT model contains several parameters that need optimization, novel optimization techniques such as the Human Felicity Algorithm (HFA), Gray Wolf Optimization (GWO), and Particle Swarm Optimization (PSO) combined with GBRT might capture complex relationships and interactions within the data more effectively. Additionally, the database used to build our prediction model contains 238 experimental results. Expanding this database to include more diverse datasets from various sources and recent experiments could improve the model’s generalizability and accuracy. Future studies should aim to continuously update and refine the database with new experimental data. Finally, future research should also explore the incorporation of novel FRP materials and advanced reinforcement techniques. Recent studies, such as those by [10,97], have shown promising results in mitigating traditional FRP failure mechanisms. Expanding the current GBRT model to include these novel materials and methods could provide deeper insights and more robust predictions.

## 6. Conclusions

Structural engineers face a major challenge in predicting the shear strength of FRP-reinforced flat concrete slabs, which underscores the need for reliable and accurate ML algorithms. Using a database of 283 FRP flat slab models, this paper proposes a gradient-boosted regression tree (GBRT) model to predict the shear strength of FRP-reinforced flat concrete slabs. The input space for the present dataset consists of seven basic parameters: cross-sectional area of the column, critical cross-sectional perimeter in the RC slab at k = 1 and k = 3, effective depth of the level slab, compressive strength of concrete, FRP longitudinal reinforcement ratio, and modulus of elasticity. The model assessment employs 5-fold cross-validation and statistical measures. An overview of the study’s conclusions and observations is provided in this section:

Based on the fivefold cross-validation results, the GBRT model demonstrated consistency and reliability across the performance metrics used in this analysis.

The higher prediction accuracy of the GBRT was demonstrated by its better performance in terms of three specified metrics (i.e., Mean Absolute Error (MAE), root mean square error (RMSE), and coefficient of determination (R^2^)) when compared to the KNN and LASSO models. Furthermore, compared to the LASSO model, the KNN model typically produced superior predictions.

Using an experimental database, the GBRT model was compared to several well-known equations, including Ospina et al. [11], El-Gamal et al. [37], El-Ghandour et al. [19], and Ju et al. [25]. The results indicated that the GBRT model provided more accurate results than the other models. The GBRT model exhibited a reduced standard deviation, coefficient of variation, and average for a given χ value when compared to the other formulations.

The perimeter of the critical section was the most influential of the seven input factors in determining the punching shear resistance of FRP flat slabs without shear reinforcement. Additionally, the punching shear prediction of the FRP flat slabs was significantly impacted by the slab effective depth. 

Based on the GBRT model, a graphical user interface was created to forecast punching shear strength, offering a useful platform for the practical design of FRP flat slabs.

## Figures and Tables

**Table 1:** Statistical measures of variables.

Statistics	*A* (cm^2^)	*b*_0,0.5*de*_ (mm)	*b*_0,1.5*de*_ (mm)	De (mm)	*f_c_* (MPa)	*ρ_r_* (%)	*E_r_* (GPa)	Vu (kN)
Mean	671.10	1496.90	2509.18	127.89	44.72	0.94	74.44	380.13
Median	625.00	1480.00	2456.00	130.00	40.00	0.89	56.70	269.50
Maximum	1587.50	2470.00	4608.00	284.00	179.00	3.76	230.00	1600.00
Minimum	6.25	280.00	640.00	36.00	22.16	0.13	28.40	24.00
Standard deviation	457.14	578.28	930.48	48.92	19.94	0.60	41.00	306.31

**Table 2:** Results derived from a five-fold cross-validation.

	Performance Measures		
Fold	MAE	RMSE	R^2^
Fold 1	85.971	127.5212	0.8614
Fold 2	44.5853	61.1113	0.9303
Fold 3	60.938	84.0522	0.9467
Fold 4	68.8669	114.5359	0.8809
Fold 5	74.898	120.6918	0.8596
Average	67.0519	101.5825	0.8958
Std deviation	15.4718	27.2149	0.0369

**Table 3:** Performance metrics of different models on training, testing, and total datasets.

Subset	Model	R^2^	RMSE	MAE
Training	GBRT	0.9634	58.8937	37.431
	KNN	0.9334	79.4677	46.5368
	Lasso	0.6969	169.5705	122.8655
Testing	GBRT	0.9188	84.4012	64.4838
	KNN	0.8446	116.7559	78.5972
	Lasso	0.6404	177.6072	135.3977
Total	GBRT	0.955	64.8508	42.887
	KNN	0.9166	88.2654	53.0028
	Lasso	0.6862	171.2217	125.393

**Table 4:** Evaluation of existing and proposed models based on χ.

Model	Mean	STD	COV
Proposed GBRT	1.0430	0.2177	0.2087
Ospina et al. [11]	1.2220	0.6841	0.5598
El-Gamal et al. [37]	0.9639	0.5760	0.5976
El-Ghandour et al. [19]	0.9820	0.5206	0.5302
Ju et al. [25]	0.9708	0.5433	0.5597

STD: standard deviation; COV: coefficient of variation.

**Table 5:** Safety classification and corresponding penalties based on χ values.

Range	Classification	Penalty (PEN)
χ > 2	Extra dangerous	10
1.15 < χ ≤ 2	Dangerous	5
0.85 < χ ≤ 1.15	Appropriate safety	0
0.5 < χ ≤ 0.85	Conservative	1
χ ≤ 0.5	Extra conservative	2

**Table 6:** Performance comparison of different models based on ratio categories and demerit points.

Category	Ju et al. [25]	El-Ghandour et al. [19]	El-Gamal et al. [37]	Ospina et al. [11]	GBRT
Ratio ≥ 2	11 (4.62%)	10 (4.2%)	11 (4.62%)	24 (10.08%)	1 (0.42%)
1.15 ≤ ratio < 2	36 (15.13%)	43 (18.07%)	37 (15.55%)	68 (28.57%)	54 (22.69%)
0.85 ≤ ratio < 1.15	61 (25.63%)	62 (26.05%)	63 (26.47%)	78 (32.77%)	155 (65.13%)
0.5 ≤ ratio < 0.85	119 (50.0%)	109 (45.8%)	117 (49.16%)	63 (26.47%)	26 (10.92%)
Ratio < 0.5	11 (4.62%)	14 (5.88%)	10 (4.2%)	5 (2.1%)	2 (0.84%)
Total demerit point score	4.31	4.52	4.32	6.53	3.1

## Data Availability

Dataset available on request from the authors.
